# Mass Spectrometric Analysis of Whole Secretome and Amylase-precipitated Secretome Proteins from *Streptococcus gordonii*

**DOI:** 10.4172/jpb.1000331

**Published:** 2014-09-04

**Authors:** A Maddi, EM Haase, FA Scannapieco

**Affiliations:** 1 Department of Oral Biology, School of Dental Medicine, State University of New York at Buffalo, Buffalo, New York, USA; 2 Periodontics and Endodontics, School of Dental Medicine, State University of New York at Buffalo, Buffalo, New York, USA

**Keywords:** Amylase-binding proteins, Proteomic analysis, Secretome, AbpA, AbpB, GtfG

## Abstract

Oral biofilm (dental plaque) is formed by the initial adhesion of “pioneer species” to salivary proteins that form the dental pellicle on the tooth surface. One such pioneer species, *Streptococcus gordonii*, is known to bind salivary amylase through specific amylase-binding proteins such as amylase-binding protein A (AbpA). Recent studies have demonstrated that once bound, salivary amylase appears to modulate gene expression in *S. gordonii*. However, it is not known if this amylase-induced gene expression leads to secretion of proteins that play a role in plaque biofilm formation. In this study we examined the differences in secreted proteomes between *S. gordonii* KS1 (wild type) and AbpA-deficient (ΔAbpA) strains. We also examined the differentially precipitated secretome proteins following incubation with salivary amylase. The culture supernatants from KS1 and ΔAbpA were analyzed by nano-LC/MS/MS to characterize the whole secreted proteomes of the KS1 and ΔAbpA. A total of 107 proteins were identified in the KS1 and ΔAbpA secretomes of which 72 proteins were predicted to have an N-terminal signal peptide for secretion. Five proteins were differentially expressed between the KS1 and ΔAbpA secretomes; AbpA and sortase B were expressed exclusively by KS1, whereas Gdh, AdcA and GroEL were expressed only by ΔAbpA. Incubation of culture supernatants from KS1 and ΔAbpA with amylase (50 μg/ml) at room temperature for 2 h resulted in the differential precipitation of secretome proteins. Hypothetical protein (SGO_0483), cation-transporting ATPase YfgQ (Aha1), isocitrate dehydrogenase (Icd), sortase A (SrtA), beta-N-acetylhexosaminidase (SGO_0405), peptide chain release factor 1(PrfA) and cardiolipin synthase (SGO_2037) were precipitated by amylase from the KS1 culture supernatant. Among the identified secreted proteins and amylase-precipitated proteins, transcriptional regulator LytR (SGO_0535) and cation-transporting ATPase YfgQ (Aha1) are potential signaling proteins.

## Introduction

*Streptococcus gordonii* belongs to the viridans group streptococci, which includes the mitis, salivarius and anginosus groups. These streptococci are essential for establishment of oral biofilms (dental plaque) and are associated with a healthy oral environment [1-3]. *S. gordonii* is one of several bacteria known to be pioneer colonizers of newly erupted teeth and cleaned tooth surfaces [4]. The formation of dental plaque initiates with the interaction between early colonizing oral bacteria and salivary components [5]. Such interactions influence the binding of other oral plaque bacteria to tooth surfaces, the aggregation of bacteria to prevent adhesion to the oral surfaces, as well as the effect of antimicrobials on the bacteria within the dental plaque [5].

Amylase is one of the most abundant proteins present in saliva. It is an enzyme that is involved in the digestion of starch to glucose, maltose and maltodextrins, which act as nutritional substrates for oral plaque bacteria. Besides its enzymatic activity, amylase adsorbs to enamel surfaces and also interacts with the pioneer colonizers [6,7]. Amylase is known to bind to the surface of S. gordonii, Streptococcus mitis, Streptococcus cristatus, Streptococcus parasanguinis, and *Streptococcus salivarius* and several other streptococci, which are collectively termed as amylase-binding streptococci (ABS) [8]. The binding and interaction of amylase with *S. gordonii* has been investigated most extensively.

*S. gordonii* produces two amylase-binding proteins (ABPs)-AbpA and AbpB [9,10]. AbpA (20 kDa) is an extracellular, cell wall-associated protein that is expressed at maximal levels during mid-log phase of bacterial growth [9]. AbpB is an 82-kDa extracellular dipeptidyl-peptidase that co-precipitates with AbpA and GtfG from culture supernatants when incubated with salivary amylase [11-13] and binds amylase in the amylase-ligand overlay assay [14]. It is clear though that AbpA is the primary receptor for amylase binding to *S. gordonii* since inactivation of AbpA essentially eliminates amylase binding to the cell surface [15,16]. However, the exact function of AbpA with regards to binding amylase is not clear. Recent studies have shown that amylase binding can induce gene expression in *S. gordonii* [16]. Since the binding of amylase to *S. gordonii* is AbpA dependent, the hypothetical question arises whether AbpA could act as a signaling receptor for salivary amylase-induced gene expression. This led us to investigate the secretome proteins and the amylase precipitated proteins to identify candidate proteins that interact with AbpA.

Although AbpA and AbpB of *S. gordonii* Challis are the predominant and best studied ABPs, SDS-PAGE analysis of amylase-precipitated proteins indicates the presence of other as yet uncharacterized proteins that appear to interact with amylase [12,17]. The secretome, which is the set of proteins secreted to the cell surface and extracellular space, has not been previously described for *S. gordonii*. Proteins of the secretome may contain signaling proteins that interact with ABPs to influence biofilm formation or interact with salivary components to induce bacterial cell signaling. In addition, comparison of differentially secreted proteins between wild type KS1 and AbpA-deficient (ΔAbpA) strains may also provide insight into the role of AbpA in *S. gordonii*. In this study, we performed 1-D electrophoresis followed by nano-liquid chromatography/mass spectrometry/mass spectrometry (nano-LC/MS/MS) analysis to identify the secretome proteins and the amylase-precipitated proteins of *S. gordonii*.

## Materials and Methods

### Preparation of secretome proteins

*S. gordonii* Challis CH1 (wild type), KS1 (kanamycin-resistant wild type), ΔAbpA (KS1Ω*abpA*) and ΔAbpB (KS1Ω*abpB*) strains were grown on tryptic soy agar supplemented with 0.5% yeast extract (TSBY) agar plates with appropriate antibiotic selection for 2-3 days at 37°C in a candle jar, as previously described [18]. For secretome preparation, isolated bacterial colonies were inoculated into 10 ml of TSBY broth and cultured overnight (12-14 h) at 37°C in a candle jar. After centrifugation at 5,000×*g*, the supernatant was collected and filtered using a 0.22-μm microfilter (Corning Inc., Corning, NY) to remove residual bacterial cells. To precipitate proteins, the filtered supernatant was transferred to a clean glass bottle and mixed well with an equal volume of 25% (w/v) trichloroacetic acid (TCA)/acetone. The samples were incubated at −20°C for at least 48 h and then centrifuged at 8,000×*g* for 10 min at 4°C. After removing the supernatant, the pellets were washed twice with 10 ml of ice-cold acetone. Upon completion of the final centrifugation, the pellets were left in a fume hood for 1-2 h to allow residual acetone to evaporate. The pellets were solubilized in 1% SDS and boiled for 10 min to denature the proteins. Protein in the samples was quantified using the BioRad DC Protein assay (Hercules, CA). Samples containing 15 μg of protein were prepared in 4× loading buffer (LDS buffer, Invitrogen, Carlsbad, CA), loaded onto a 10% SDS-PAGE gel (NuPage gels, Invitrogen, Carlsbad, CA), and resolved by electrophoresis. Gels were stained using the SilverQuest silver staining kit (Invitrogen, Carlsbad, CA). For MS/MS analysis, the samples (15 μg protein/lane) were loaded onto a 10% SDS-PAGE gel and subjected to gel electrophoresis briefly for 5-10 min until the samples completely entered the resolving gel. Electrophoresis was stopped and the gel was stained with Coomassie blue. The single stained band in each lane containing the whole “secreted proteome” was cut from the gel and sent for nano-LC/MS/MS analysis (Fred Hutchinson Cancer Research Center, Seattle, WA). This experiment was repeated from two independent biological samples.

#### In-gel trypsin digestion

The gel pieces were destained with 25 mM ammonium bicarbonate in 50% acetonitrile, and subsequently dehydrated using acetonitrile. The proteins were digested overnight with 5 ng/μL trypsin (Promega Corporation) in 50 mM ammonium bicarbonate at 37°C. The peptides were extracted from the gel using 0.1% (v/v) trifluoroacetic acid (TFA) in water after 30 min incubation followed by acetonitrile. The pooled extracts were dried in a SpeedVac and cleaned using ZipTip™ C18 (Millipore Corporation) before MS analysis.

#### Nano-LC-MS/MS analysis

LC-MS/MS analysis was performed using a LTQ Orbitrap XL mass spectrometer (Thermo Scientific). The LC system configured in a vented format consisted of a fused-silica nanospray needle packed in-house with Magic C18 AQ 100A reverse-phase media (Michrom Bioresources Inc, 25 cm) and a trap (2 cm) containing Magic C18 AQ 200A reverse-phase media. The inner diameters of the analytical and trap columns were 75 μm and 100 μm, respectively. The peptide samples were loaded onto the column and chromatographic separation was performed using a two-mobile-phase solvent system consisting of 0.1% formic acid in water (A) and 0.1% acetic acid in acetonitrile (B) over 60 min from 5% B to 40% B. The mass spectrometer operated in a data-dependent MS/MS mode over the *m/z* range of 400-1800. For each cycle, the five most abundant ions from each MS scan were selected for MS/MS analysis using 35% normalized collision energy. Selected ions were dynamically excluded for 45 seconds.

#### Data analysis

Raw MS/MS data were submitted to the Computational Proteomics Analysis System (CPAS), a web-based system built on the LabKey Server v11.2 and searched using the X!Tandem search engine against *S. gordonii* Challis protein database from UniProt (http://www.uniprot.org/). The search output files were analyzed and validated by ProteinProphet. Peptide hits were filtered with PeptideProphet error rate <0.05, and proteins with probability scores of >0.9 were accepted.

### Amylase precipitation of proteins from culture supernatant

*S. gordonii* KS1 and ΔAbpA were grown on TSBY agar with appropriate antibiotic selection for 2-3 days at 37°C in a candle jar. Isolated bacterial colonies were inoculated into 10 ml of TSBY broth and cultured overnight (12-14 h) at 37°C in a candle jar. After centrifugation, the culture supernatant was collected and filtered using a 0.22-μm microfilter. Proteins were precipitated with salivary amylase as described previously [16]. Briefly, purified human salivary amylase (50 μg/ml), was added to the filtered supernatant in a sterile tube and incubated statically at room temperature for 2 h followed by centrifugation at 5,000×*g* for 10 min. After carefully removing the supernatant, the pellet containing the precipitated proteins was solubilized in 1% SDS and boiled for 10 min to denature the proteins. The protein content of the samples was quantified using the BioRad DC Protein assay. Samples of equal protein content (15 μg) were prepared for electrophoresis in 4X loading buffer, loaded onto 10% SDS PAGE gels (NuPage gels, Invitrogen), and subjected to electrophoresis. The gels were silver stained to visualize protein separation. For MS/MS analysis, a similar gel was stained with Coomassie blue and four bands were cut above and one band below the amylase band from both the KS1 and AbpA samples, as shown in Figure 3. Bands were sent for nano-LC/MS/MS analysis (Applied Biomics, Inc, Hayward, CA). As a control, concentrated TSBY was sent to determine potential growth medium derived proteins in the samples. This experiment was repeated from two independent biological samples.

#### Reduction/alkylation and trypsin digestion

DTT was added to a final concentration of 10 mM in 50 mM ammonium bicarbonate and incubated with the gel band at 60°C for 30 min, followed by cooling down to room temperature. Iodoacetamide was then added to a final concentration of 50 mM and incubated in the dark for 30 min at room temperature. The gel band was washed a few times and then digested in-gel with modified porcine trypsin protease (Promega).

#### NanoLC-MS/MS

NanoLC was carried out using a Dionex Ultimate 3000 (Milford, MA). Tryptic peptides were loaded into a μ-Precolumn Cartridge and separated on an acetonitrile gradient (ranging from 5% to 60%) on a Nano LC column. Fractions were collected at 20-second intervals followed by Mass Spectrometry analysis on AB SCIEX TOF/TOF™ 5800 System (AB SCIEX). Mass spectra were acquired in reflectron positive ion mode. TOF/TOF tandem MS fragmentation spectra were acquired for each ion, averaging 4000 laser shots per fragmentation spectrum (excluding trypsin autolytic peptides and other known background ions).

#### Data analysis

Both of the resulting peptide mass and the associated fragmentation spectra were submitted to GPS Explorer workstation equipped with MASCOT search engine (Matrix Science, London, UK) to search the database of National Center for Biotechnology Information non-redundant (NCBInr). Searches were performed without constraining protein molecular weight or isoelectric point, with variable carbamidomethylation of cysteine and oxidation of methionine residues, and with one missed cleavage also allowed in the search parameters.

### Bioinformatics

A bioinformatics approach was used to further characterize the proteins for molecular weight, putative functions and domains. To determine molecular weight, FASTA amino acid sequences were obtained from the *S. gordonii* Challis database at National Center for Biotechnology Information (NCBI) and submitted to http://web.expasy.org/compute_pi/. For prediction of N-terminal signal peptides for secretion, the FASTA amino acid sequences were submitted to http://www.predisi.de/home.html. To determine functional domains, the FASTA amino acid sequences were submitted to the protein BLAST at NCBI.

## Results and Discussion

### Whole secretome analysis

The secretome of CHI, KS1, ΔAbpA and ΔAbpB, comprising both secreted and cell-associated proteins shed during pellet preparation, were compared. To extend the results of our previous work [11,12], bacteria were grown in nutrient-rich TSBY medium to stationary phase (12-14 h). The use of rich medium avoids the secretion of proteins induced under the stress of nutrient limitation in defined medium. AbpA is secreted throughout the growth phase, whereas AbpB is expressed from late exponential to stationary phase [9], thus examination of the secretome of cells in stationary phase enabled the study of both known ABPs of *S. gordonii*, as well as other potentially associated secreted proteins.

A silver stained SDS-PAGE gel of TCA-precipitated whole secretome samples revealed only minor differences in protein band patterns between CH1 (wild type), KS1 (kanamycin-resistant wild type) and ΔAbpA strains. Although these differences appeared to be minor from the stained protein gels, the presence of a large number of closely spaced bands makes their differentiation difficult. Hence, a mass spectrometric (nano-LC/MS/MS) analysis of the whole secretome samples was performed to identify proteins within each sample. The protein sample was treated with trypsin to generate multiple peptides. Mass spectrometry (MS) allows for differential identification of peptides present in the samples. Differential expression between wild-type and mutant strains is based on presence or absence of the protein-associated peptides in the secretome preparations. There were no differences evident between the CH1 and KS1 secretomes (data not shown) supporting the observations by SDS-PAGE analysis. The TSBY media contains yeast extract, which may contribute to the peptide sequences in the MS analysis. For this reason an MS analysis of TCA precipitate of the TSBY media was performed and no yeast proteins were found (data not shown). Concurrently, in any of the MS analyses for secreted proteomes yeast proteins were not found, however, human keratin was found as a contaminant.

The whole secretomes of KS1 and ΔAbpA were compared. A total of 107 proteins were identified (Tables 1 and 2). This is the first study to identify whole secretome proteins from *S. gordonii* using nano-LC/MS/MS analysis. The secretome fractions include several classically secreted proteins with N-terminal signal peptides, secreted proteins with enzymatic domains and non-canonical secretory proteins, which mostly include cytosolic proteins. Several hypothetical proteins of unknown functions and uncharacterized domains were also identified.

It is notable that the majority of 107 proteins contained an N-terminal signal peptide. In fact, 72 proteins (67%) were predicted to have an N-terminal signal peptide for secretion typical for classically secreted proteins. The other 35 proteins (33%), which did not contain an N-terminal signal peptide, were likely of cytosolic origin or may be considered as non-classical secreted proteins (Table 1). Among the classically secreted proteins predicted to be cell wall surface associated, 11 proteins contained a consensus C-terminal motif Leu-Pro-X-Thr-Gly (LPXTG) cell wall anchor motif and 9 proteins lacked this motif.

**Figure 1:**
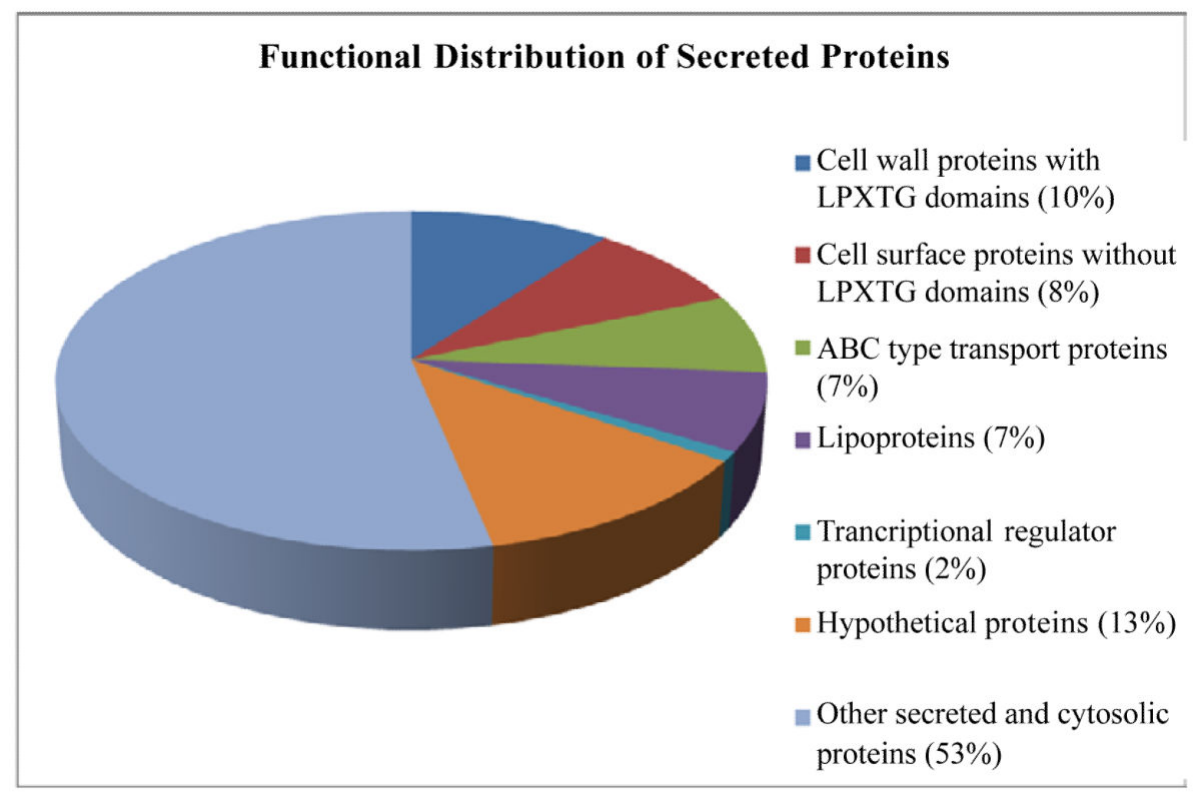
Functional distribution of proteins in whole secretome of *Streptococcus gordonii*.

The majority of proteins that have been identified in the secretome of *S. gordonii* represent proteins observed in the stationary phase of other gram-positive bacteria. Even in rich medium during the stationary phase of growth nutrient limitation and decrease in pH can trigger various stress responses [19]. In *Streptococci pyogenes*, transition to early stationary phase was associated with acidification of the growth medium to approximately pH 5.5 and a depletion of glucose [20]. Stationary phase cytoplasmic proteins of *S. pyogenes* grown in Todd-Hewitt broth showed an abundance of enzymes involved in glycolysis and pyruvate metabolism, as well as stress-responsive proteins [21]. An increase in enzymes in central metabolism is thought to enhance the ability to scavenge carbohydrates [22]. Thus, proteins associated with energy production, carbohydrate transport, amino acid transport, lipid transport, nucleotide transport, cell wall biogenesis, protein turnover, and posttranslational modification are expected to be more abundant. In this study, during the stationary phase of growth, we found a significant number of secreted proteins, lipoproteins, ABC type transporter proteins and proteins related to cell wall biogenesis as part of the secretome. The overall functional distribution of the secretome proteins is listed in Figure 1.

Consistent with the silver stained gel, a total of 102 proteins were found in common to both the KS1 and ΔAbpA secretomes (Table 1). Only five proteins were found to be consistently differentially expressed based on the analyses of secretome samples from two independent experiments (Table 2). AbpA and sortase B were identified in the KS1 secretome fraction by LC-MS/MS analysis, whereas they were absent in the ΔAbpA strain. AbpB and GtfG were present in both strains indicating that their secretion was not affected by inactivation of *abpA*. Glutamate dehydrogenase (SGO_0276), metal-binding (Mn) permease lipoprotein (SGO_1936), and the 60-kDa chaperonin (groEL) (SGO_1885) were identified in the ΔAbpA secretome while they were absent in the KS1 strain (Table 2).

AbpA and sortase B (SrtB) were differentially expressed among the secretome proteins in KS1 and ΔAbpA. The absence of AbpA protein in the secretome of ΔAbpA confirms its inactivation. SrtB, a transpeptidase that anchors proteins to the cell wall peptidoglycan through a NXZTN sorting motif [23], was also absent in the secretome of ΔAbpA. The *srtB* gene locus is downstream of *abpA* in the *S. gordonii* Challis (CH1) genome. The absence of SrtB in the secretome of ΔAbpA suggests co-transcription of *abpA* and *srtB*. This is also supported by recent transcriptome analysis, which showed that *srtB* was significantly downregulated in ΔAbpA grown to mid-log phase in TSBY (unpublished data). Considering that *abpA* and *srtB* may be transcribed as a polycistronic message, the insertion of the tetracycline resistance gene *tet*(M), which contains a weak terminator, into *abpA* may have caused a polar effect on the downstream *srtB* gene.

Other differentially secreted proteins, including glutamate dehydrogenase, metal-binding (Mn) permease lipoprotein AdcA (SGO_1936), and chaperonin (GroEL) were identified in ΔAbpA but were absent in KS1. These proteins are non-classical secreted proteins, as they lack the N-terminal signal peptide targeting a protein for secretion. Each of these proteins has been shown to play a role in resistance to stress in various organisms. Glutamate dehydrogenase is a housekeeping enzyme in bacteria that functions in nitrogen metabolism as part of the urea cycle by converting glutamate to oxoglutarate. It is usually found in the cytoplasm and the cell membrane. It has been shown that glutamate dehydrogenase functions in providing resistance to stress in yeast cells [24]. In *Clostridium difficile*, extracellular glutamate dehydrogenase was found to confer resistance to hydrogen peroxide [25]. Metal-binding (Mn) permease lipoproteins may act as surface ligands for host cell matrix proteins [26]. Also, metal-binding (Mn) permease lipoprotein/AdcA belongs to the TroA superfamily of metal binding lipoproteins and may be involved in the sequestration of metal ions like manganese. A TroA knockout in *Streptococcus suis* resulted in manganese deprivation and reduced virulence in a mouse model [27]. Susceptibility of the TroA mutant to hydrogen peroxide indicated that TroA could be playing a role in oxidative stress [27]. The role of AdcA in *S. gordonii* remains to be determined.

GroEL is a 60-kDa chaperonin protein that is involved in protein folding in bacterial secretion systems [28]. GroEL is highly conserved among bacterial species and is also used for differentiating various streptococci [29]. Interestingly, it is also a protein that is upregulated along with DnaK under general stress response [30]. GroEL functions to allow unfolded proteins produced by stress to fold and prevent aggregation with other proteins [31]. It is frequently found in secretomes of other bacteria under various stress conditions such as increased oxygen tension and acidic pH [32-34]. It is possible that AbpA may have functions independent of amylase binding that may elicit changes in response to stress.

### Amylase-precipitated proteins

**Figure 2:**
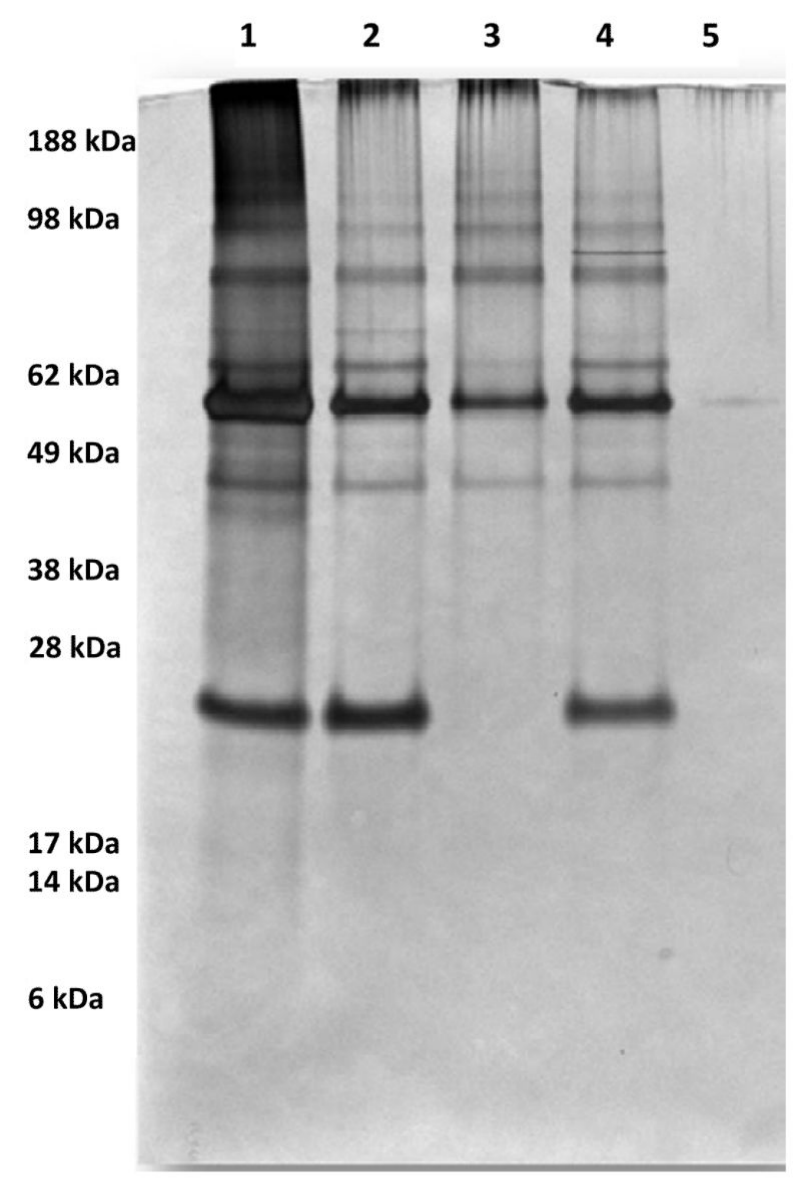
Silver stained 10% SDS-PAGE gel showing amylase-precipitated proteins in various strains of *S. gordonii* strains Lanes: 1-CH1 (WT), 2-KS1 (kanamycin resistant WT), 3-ΔAbpA, 4-ΔAbpB and 5-amylase (control).

**Figure 3:**
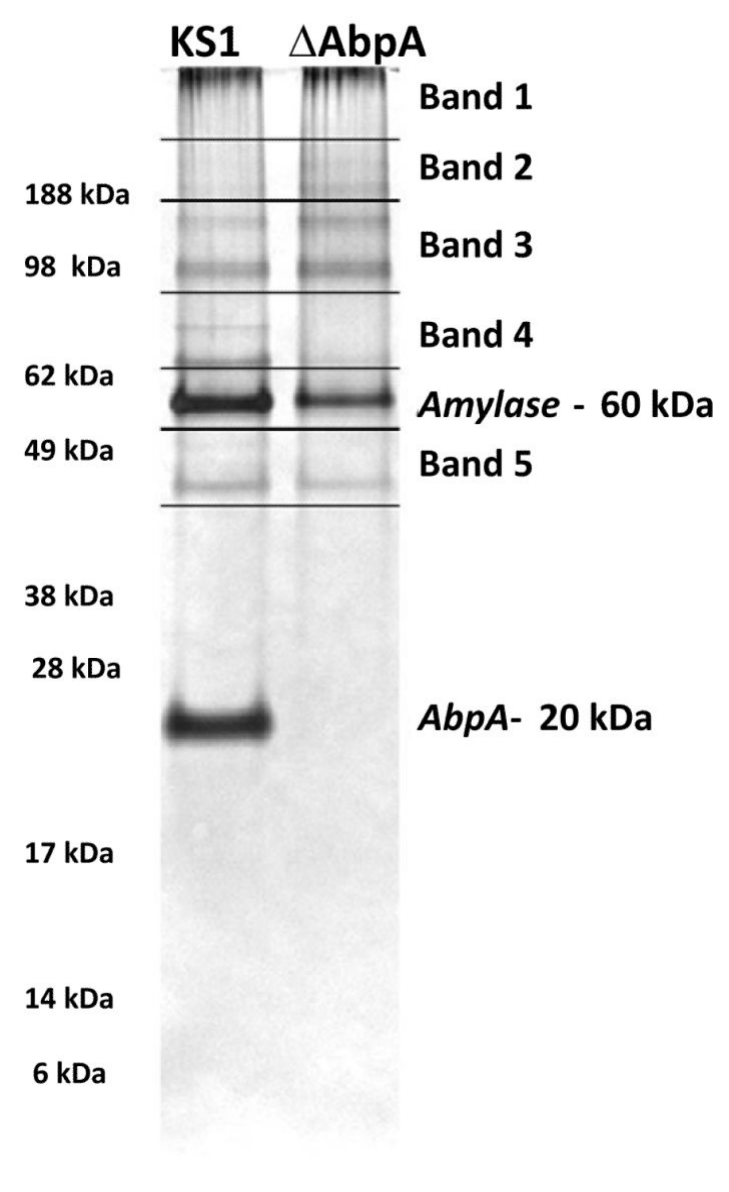
Sample preparation for MS/MS analysis of amylase-precipitated proteins Coomassie-stained gels were cut in the pattern depicted above to get 5 bands/slices per strain. Amylase and AbpA bands were avoided as they are well known from past experiments. The gel bands/slices were placed in microcentrifuge tubes containing 70 μl of double distilled water and sent for nano-LC/MS/MS analysis.

Previous studies in our lab identified *S. gordonii* proteins that interacted with salivary amylase. AbpA (20 kDa), AbpB (82 kDa) and glucosyltransferase G (GtfG; 174 kDa) were the predominant proteins precipitated by amylase from culture supernatants of wild-type strains based on SYRO Red staining of 12% SDS-PAGE gels and MALDI-TOF analysis of eluted proteins [11,12]. In the current study, an additional 5-6 protein bands were revealed by silver stain in the amylase-precipitated protein samples (Figure 2). Silver staining is very sensitive and aids in visualizing proteins even at picogram levels. To identify these proteins, bands were sliced from a duplicate Coomassie-stained gel (Figure 3), as described above, and subjected to nano-LC/MS/MS-analysis. Based on the MS analysis, several proteins were identified in each of the sliced gel bands.

The amylase-precipitated proteins identified in KS1 and ΔAbpA are listed in Table 3. AbpA was excluded from the analysis, as no bands were sliced below 40 kDa. Interestingly AbpB and GtfG were not detected in the amylase precipitates of KS1 contrary to previous studies [11,12]. This could be due to several reasons. Growth of cultures to late stationary phase could have degraded some AbpB and GtfG resulting in very low quantities to be precipitated by amylase. Also, the abundant presence of several other amylase-binding proteins could have out-competed the binding of AbpB and GtfG. This is evident by the fact that both AbpB and GtfG were identified by mass spectrometric analysis of TCA-precipitated whole secretome protein fractions. The method of detection can also have a major influence on what is detected. The method of detection in our previous analysis is significantly different from the methods used in this study [11,12]. Previously, after amylase precipitation, the whole protein pellet was solubilized in water and run on a 12% SDS PAGE gel and stained with SYPRO Red. AbpA and GtfG were then identified by N-terminal sequencing of blots. In this study, the resulting pellet following amylase precipitation was solubilized in 1% SDS and boiled for 10 mins. Proteins were quantified after adding SDS containing buffer with a detergent compatible protein assay. Only 10-15 μg protein per lane was loaded on 10% SDS-PAGE gels and silver staining was performed for visualization. Duplicate gels were Coomassie stained and gel slices were submitted for MS analysis. Furthermore, as previously demonstrated, it is not necessary that all of the identified amylase-precipitated proteins bind directly to amylase. GtfG was absent from amylase-precipitated culture supernatants from the AbpA-deficient strain, suggesting that AbpA interacted with and precipitated GtfG [12]. Likewise, the primary proteins like AbpA and AbpB could be bound to amylase and the remaining proteins could be bound to these primary proteins forming a complex of amylase precipitated proteins. Considering that a large number of proteins are potentially part of this amylase-precipitated complex, it is to be determined as to what their exact role would be.

Some of the other amylase-precipitated proteins were expressed in the KS1 strain, but absent in the ΔAbpA strain (Table 3). No proteins unique to ΔAbpA were identified. The classically secreted proteins differentially expressed in KS1 included: cation-transporting ATPase YfgQ (T3 secretion) (SGO_1458), sortase A (SGO_1230), and beta-N-acetylhexosaminidase (SGO_0405). The non-classical secreted proteins identified in KS1 included: hypothetical protein (SGO_0483), isocitrate dehydrogenase (SGO_1611), and cardiolipin synthase (SGO_2037). YfgQ is a putative protein with a conserved cation-transporting ATPase domain whose exact function is not known. Sortase A is an enzyme that is involved in cross-linking proteins to the cell wall in gram-positive bacteria. Sortase A recognizes the LPXTG motif present at the N-terminal of cell wall proteins and anchors them to the cell wall [35]. Beta-N-acetylhexosaminidases of glycosyl hydrolase family 20 (GH20) catalyze the removal of beta-1,4-linked N-acetyl-D-hexosamine residues from the non-reducing ends of N-acetyl-beta-D-hexosaminides including N-acetylglucosides and N-acetylgalactosides. These enzymes are broadly distributed in microorganisms, plants and animals, and play roles in various key physiological and pathological processes [36].

The non-classical secreted proteins-hypothetical protein (SGO_0483), isocitrate dehydrogenase (SGO_1611), and cardiolipin synthase (SGO_2037) were identified in the KS1 strain and were absent in the ΔAbpA strain. Isocitrate dehydrogenase catalyzes the oxidative decarboxylation of isocitrate to alpha-ketoglutarate and can use either NAD(+) or NADP(+) as a cofactor. Recent studies demonstrate that the NADP(+)-dependent isocitrate dehydrogenase, as a source of electrons for cellular antioxidants, is important for protection against oxidative damage [37]. Cardiolipin synthase has an important role in modulating the physical properties of membranes in response to environmental changes. It promotes formation of membrane subdomains that can serve to localize and regulate assembly of protein complexes, which is critical for cell division and membrane transport [38].

Most of the non-classical proteins present in either the whole secretome or those precipitated by amylase usually function in the cytoplasm. While it cannot be ruled out that some of these non-classical secreted proteins may have originated from cell lysis, they may be secreted by other mechanisms such as membrane vesicles or specific membrane microdomains [39-41]. The redox protein thioredoxin, which lacks an N-terminal signal peptide, was found here in the whole secretomes of both KS1 and ΔAbpA, as well as in the secretomes of mammals, plants, and other bacteria [42-44]. Other proteins that include glycolytic pathway enzymes like enolase, membrane associated proteins and chaperone proteins can elicit strong immunogenicity and take part in virulence and pathogenesis of disease [42]. The role of thioredoxin and other secreted proteins lacking N-terminal signal peptides in the extracellular space may be similar to the cytoplasmic function or have a yet unrecognized function.

## Summary and Conclusions

We have identified a set of more than 107 proteins in the secretome of *S. gordonii* for the first time. Some of these proteins are differentially expressed in the ΔAbpA strain as compared to the KS1 strain (wild type) indicating that AbpA may possibly regulate their expression in stationary phase. Some of these differentially expressed proteins include AdcA and GroEL, which are involved in oxidative stress response. Further studies are required to determine the extracellular role of these proteins. By using mass spectrometric analysis we were able to identify the previously uncharacterized amylase-precipitated proteins. It appears that apart from AbpA, AbpB and GtfG there are several other proteins that could bind to salivary amylase (or AbpA), although it is not clear whether this binding is direct or indirect. Future work will focus on the effect of amylase and starch on protein secretion by *S. gordonii*.

## Figures and Tables

**Table 1:** Whole secretome proteins identified in common from KS1 and ΔAbpA.

Protein/Gene Name	Gene Index	Predicted Function/Domain	Mol. Wt. (kDa)	Pep. Id.	Aa. Seq. Cov. (%)
General stress protein gsp-781*	SGO_2107	Unknown	41	211	63
Glucosyltransferase G gtfG*	SGO_0497	Glycosylhydrolase	178	175	62.8
Serine protease challisin sgc*	SGO_0566	Serine protease	165	131	52.2
Serine protease degP*	SGO_2150	Serine protease	42	102	43.6
Surface-associated protein cshA*	SGO_0854	Unknown	264	85	40.1
Streptococcal surface protein A sspA*	SGO_0210	Unknown	172	75	38.3
Surface antigen SCP-like domain	SGO_1110	Unknown	82	72	46.2
Membrane protein	SGO_0060	Membrane protein	109	66	41.8
5′-nucleotidase family protein*	SGO_1247	N-terminal metallophosphatase domain	78	64	55.6
Streptococcal surface protein B sspB*	SGO_0211	Unknown	164	58	38.7
LPXTG cell wall surface protein*	SGO_0707	Cell wall surface protein	180	52	54.6
LPXTG cell wall surface protein*	SGO_1651	Cell wall surface protein	84	50	37.9
Surface-associated protein cshB*	SGO_1148	Unknown	244	46	32.9
Penicillin-binding protein 1B pbp1B	SGO_1928	Transpeptidase domain	92	34	43.6
Exo-beta-D-fructosidase*	SGO_0385	Glycosyl hydrolases family 32	156	32	29.9
LPXTG cell wall surface protein*	SGO_0890	Collagen binding domain	69	32	22.7
Zinc metalloproteinase B zmpB*	SGO_0408	IgA1-specific Metallo-endopeptidase N-terminal region	218	28	18.2
Putative transcriptional regulator lytR* ∞	SGO_0535	Membrane bound transcriptional regulator	44	28	44.2
Lipoprotein*	SGO_1082	Lipoprotein	37	28	70.2
LPXTG cell wall surface protein*	SGO_1487	Cna protein B-type domain	191	28	50.7
Putative N-acetylmuramidase*	SGO_2013	Glycosyl hydrolases family 25	127	28	33.4
Lipoprotein*	SGO_0094	Unknown	42	26	43.1
Putative uncharacterized protein	SGO_1066	Unknown	49	26	53.9
Conserved domain protein*	SGO_0021	Unknown	20	25	60.4
Putative uncharacterized protein*	SGO_2136	Unknown	48	25	47.2
Putative uncharacterized protein	SGO_0319	Unknown	32	23	26.1
LysM domain protein*	SGO_0138	Involved in binding peptidoglycan	40	21	17.7
Oligopeptide-binding protein*	SGO_1716	ABC-type oligopeptide transport system	73	20	35.7
LPXTG cell wall surface protein*	SGO_0388	Zinc carboxypeptidase	120	19	22.3
LPXTG cell wall surface protein*	SGO_0430	Unknown	96	19	35.3
Serine/threonine protein kinase	SGO_0600	Serine/threonine protein kinase	67	18	24.6
Maltose/maltodextrin-binding protein*	SGO_0104	Maltose binding domain	45	17	44.2
Oligopeptide-binding lipoprotein hppH*	SGO_1715	ABC-type oligopeptide transport system	72	17	32.2
Protein with prophage function domain	SGO_0067	Prophage function domain	52	16	35
Glyceraldehyde-3-phosphate dehydrogenase gap	SGO_0207	Glycolysis	36	15	41.1
Transport protein	SGO_0767	TroA like transporter domain	32	14	47.8
LPXTG cell wall surface protein*	SGO_1650	Collagen binding domain	77	14	18.7
Pneumococcal histidine triad A protein	SGO_1313	Unknown	124	13	13
Autolysin, N-acetylmuramidase-like protein	SGO_1138	Unknown	26	12	40.2
Sulfatase*	SGO_1377	Sulfatase	83	12	26.3
LPXTG cell wall surface protein	SGO_2005	Unknown	397	12	4.9
LPXTG cell wall surface protein*	SGO_0107	Collagen binding domain	115	11	10.9
Amylase-binding protein B abpB*	SGO_0162	Amylase-binding protein	73	11	25.5
Lipoprotein*	SGO_0181	Unknown	36	11	36.7
Cell wall binding protein*	SGO_0477	Glucan-binding domain	36	11	42.4
Peptidoglycan N-acetylglucosaminedeacetylase A pgdA*	SGO_0948	Peptidoglycan N-acetylglucosaminedeacetylase	54	11	21.1
Penicillin-binding protein 2B pbp2B	SGO_1449	Transpeptidase domain	75	11	22.8
Oligopeptide-binding lipoprotein hppG*	SGO_1713	ABC-type oligopeptide transport	74	11	28.5
Lipoprotein*	SGO_0068	Unknown	20	10	42.4
Lipoprotein hlpA*	SGO_0458	ABC-type metal ion transport system	32	9	29.6
Cell wall binding protein*	SGO_0478	Glucan-binding domain	37	9	38
Substrate-binding protein msmE*	SGO_1305	Sugar transport	49	9	35.9
Branched-chain amino acid ABC transporter*	SGO_1630	Amino acid-binding protein	41	9	25.3
Conserved domain protein*	SGO_0018	Transglycosylase-like domain	21	8	25.5
Elongation factor Tu tuf	SGO_0761	Protein translation	44	7	23.4
Enolase eno	SGO_1426	Glycolysis	47	7	17.1
Cell wall binding protein*	SGO_0845	Glucan-binding domain	34	7	12.8
Cell shape-determining protein mreC*	SGO_2108	Rod shape-determining protein	31	7	29.6
Penicillin-binding protein 1A pbp1a*	SGO_0586	Penicillin binding protein transpeptidase domain	79	6	11.7
Putative uncharacterized protein	SGO_0591	Unknown	50	6	10.5
SCP-like extracellular protein*	SGO_0847	SCP-like extracellular protein domain	56	6	14.9
Putative uncharacterized protein	SGO_0332	Unknown	53	5	15.6
Amino acid ABC transporter*	SGO_0982	Amino acid-binding protein domain	31	5	18.7
Foldase protein prsA*	SGO_1572	PPIase domain	34	4	18.8
Penicillin-binding protein 2× pbp2×*	SGO_0575	Penicillin binding protein transpeptidase domain	83	4	5.7
Efflux transporter, RND family, MFP subunit family*	SGO_0750	RND family efflux transporter	42	4	18.3
Lipoprotein*	SGO_1165	Unknown	33	4	21.6
Sortase A srtA*	SGO_1230	Cell wall anchoring of LPXTG proteins (Nobbs, 2007)	28	4	8.8
LPXTG cell wall surface protein*	SGO_1415	X-prolyldipeptidylaminopeptidase	117	4	7.6
Peptidyl-prolylcis-trans isomerase*	SGO_1463	Peptidyl-prolylcis-trans isomerase	29	4	12.7
Metal ABC transporter substrate-binding lipoprotein*	SGO_1802	Metal ABC transporter	35	4	22.6
Elongation factor G fusA	SGO_0206	Protein translation	77	3	10.2
Chaperone protein dnaK	SGO_0402	Protein folding	65	3	4.9
L-lactate dehydrogenase ldh	SGO_1232	L-lactate dehydrogenase	35	3	9.1
Putative lipoprotein*	SGO_0004	Unknown	20	3	17.4
Possible cell wall protein*	SGO_0846	SCP-like extracellular protein domain n	76	3	5.2
Glutamine ABC transporter permease and substrate binding protein*	SGO_1037	ABC transporter	53	3	6.4
Thioredoxin family protein	SGO_1171	Thioredoxin	18	3	31.2
D-Alanyl-D-Alanine carboxypeptidase*	SGO_1585	D-alanyl-D-alanine carboxypeptidase	51	3	6.7
5′-nucleotidase, lipoprotein*	SGO_1860	Haloaciddehalogenase-like hydrolases	32	3	11.2
Putative uncharacterized protein	SGO_1870	Unknown	65	3	5.1
Putative uncharacterized protein*	SGO_1932	Unknown	22	3	24.6
Lipoprotein*	SGO_2038	Unknown	17	3	21.4
Lipoprotein*	SGO_0227	Unknown	23	3	8.1
Arginine–tRNA ligase argS	SGO_2058	Arginine–tRNA ligase	63	3	6.9
Putative peptidoglycan hydrolase and general stress protein*	SGO_0212	Putative peptidoglycan hydrolase	23	3	12
Putative uncharacterized protein pXO1	SGO_0059	Unknown	11	2	35.8
Putative uncharacterized protein	SGO_0080	Unknown	62	2	5.3
Putative acyltransferase*	SGO_0112	Putative acyltransferase	68	2	3.5
Putative uncharacterized protein*	SGO_0329	Unknown	69	2	5.4
Aminodeoxychorismatelyase-like protein	SGO_0518	Unknown	60	2	3.3
Pyruvate kinase pyk	SGO_1339	Glycolysis	55	2	7.4
ABC transporter, substrate-binding protein*	SGO_1763	ABC transporter	54	2	7.6
LPXTG cell wall surface protein, glycosyl hydrolase*	SGO_0208	Glycosyl hydrolase family 85	174	2	1.6
Lipoprotein*	SGO_1931	Unknown	19	2	9.4
ABC transporter, substrate-binding protein*	SGO_0457	ABC transporter	32	2	6.7
Putative uncharacterized protein	SGO_0832	Unknown	13	2	26.3
Penicillin-binding protein 2A pbp2A	SGO_2010	Penicillin binding protein transpeptidase domain	81	2	2.6
Penicillin-binding protein 3 pbp3*	SGO_1717	Penicillin-binding protein domain	46	2	5.6
Elongation factor Ts tsf	SGO_2000	Protein translation	37	2	7.2
Putative uncharacterized protein	SGO_1756	Unknown	19	2	16.1
Zinc proteinase*	SGO_2009	Zinc metalloprotease	34	2	7.9

**Mol. Wt. kDa** – Molecular weight in kDa (http://web.expasy.org/compute_pi/)

**Pep. Id.** – No. of peptides identified in MS/MS analysis

**Aa. Seq. Cov.** – % of the total aminoacid sequence covered by identified peptides

* Proteins with N-terminal signal for secretion (http://www.predisi.de/home.html)

**Table 2:** Differentially secreted proteins in KS1 and ΔAbpA strains.

*Protein/Gene Name*	*Gene Index*	*Predicted Function/Domain*	*Mol. Wt. kDa*	*Pep.* *Id.*	*Aa. Seq.* *Cov. (%)*	*KS1*	*ΔAbpA*
Amylase-binding protein abpA*	SGO_2105	Amylase-binding protein	20	12	53.8	+	−
Sortase B srtB*	SGO_2104	Cell surface protein anchoring	32	2	8.4	+	−
Glutamate dehydrogenase gdh	SGO_0276	Glutamate dehydrogenase	49	3	8.7	−	+
Metal binding permease adcA*	SGO_1936	Metal transport	56	2	7.2	−	+
60 kDa chaperonin groEL	SGO_1885	Chaperone protein	57	2	9.3	−	+

**Mol. Wt. kDa**–Molecular weight in kDa (http://web.expasy.org/compute_pi/)

**Pep. Id.**–No. of peptides identified in MS/MS analysis

**Aa. Seq. Cov.**–% of the total aminoacid sequence covered by identified peptides

* Proteins with N-terminal signal for secretion (http://www.predisi.de/home.html)

**Table 3:** MS/MS analysis of amylase-precipitated proteins from KS1 and ΔAbpA. Underlined proteins are differentially precipitated.

Bands	KS1	ΔAbpA
Band 1	50S ribosomal protein L5 Acetyltransferase Hypothetical protein SGO_0483 (Non- secretory)	50S ribosomal protein L5
Band 2	50S ribosomal protein L5 Acetyltransferase Argininosuccinate synthase Cation-transporting ATPase yfgQ (T3 secretion)	50S ribosomal protein L5
Band 3	50S ribosomal protein L5 Isopropylmalateisomerase large subunit Isocitrate dehydrogenase Sortase A-LPXTG cell wall surface protein Beta-N-acetylhexosaminidase (Secreted protein)	Isopropylmalateisomerase large subunit 50S ribosomal protein L5
Band 4	50S ribosomal protein L5 Argininosuccinate synthase Peptide chain release factor 1	50S ribosomal protein L5 Acetyltransferase
Band 5	50S ribosomal protein L5 General stress protein GSP-781 Cardiolipin synthase (Non-secretory) Hypothetical protein SGO_0483 (Non- secretory)	50S ribosomal protein L5 General stress protein GSP-781

## Submit your next manuscript and get advantages of OMICS Group submissions

### Unique features

- User friendly/feasible website-translation of your paper to 50 world’s leading languages
- Audio Version of published paper
- Digital articles to share and explore

### Special features

- 350 Open Access Journals
- 30,000 editorial team
- 21 days rapid review process
- Quality and quick editorial, review and publication processing
- Indexing at PubMed (partial), Scopus, EBSCO, Index Copernicus and Google Scholar etc
- Sharing Option: Social Networking Enabled
- Authors, Reviewers and Editors rewarded with online Scientific Credits
- Better discount for your subsequent articles
